# Mapping Neural Circuits with Activity-Dependent Nuclear Import of a Transcription Factor

**DOI:** 10.3109/01677063.2011.642910

**Published:** 2012-01-12

**Authors:** Kaoru Masuyama, Yi Zhang, Yi Rao, Jing W Wang

**Affiliations:** 1 Neurobiology Section, Division of Biological Sciences, University of California, San Diego, La Jolla, California, USA; 2 Peking-Tsinghua Center for Life Sciences, Peking University School of Life Sciences, Beijing, China

**Keywords:** *Drosophila*, olfaction, antennal lobe, pheromone, NFAT, activity dependent, N2A, immediate-early gene

## Abstract

Nuclear factor of activated T cells (NFAT) is a calcium-responsive transcription factor. We describe here an NFAT-based neural tracing method—CaLexA (calcium-dependent nuclear import of Lex A)—for labeling active neurons in behaving animals. In this system, sustained neural activity induces nuclear import of the chimeric transcription factor LexA-VP16-NFAT, which in turn drives green fluorescent protein (GFP) reporter expression only in active neurons. We tested this system in *Drosophila* and found that volatile sex pheromones excite specific neurons in the olfactory circuit. Furthermore, complex courtship behavior associated with multi-modal sensory inputs activated neurons in the ventral nerve cord. This method harnessing the mechanism of activity-dependent nuclear import of a transcription factor can be used to identify active neurons in specific neuronal population in behaving animals.

## INTRODUCTION

One of the main challenges in systems neuroscience is to correlate the actions of neurons, synapses, and circuits with specific behaviors. Although electrophysiological methods are capable of monitoring neural activity with unparalleled resolution, they are laborious and difficult to apply to large groups of neurons. Among the alternatives, genetically encoded reporters of neural activity in optical imaging are an attractive choice over chemical probes, because they eliminate the nontrivial problem of loading chemicals into specific cells. The last decade has seen the widespread use of neural activity reporters that track vesicle release at axon terminals (Miesenbock et al., 1998) and intracellular ion concentrations (Kuner & Augustine, 2000; Mank et al., 2008; Miyawaki, 2003; Nakai et al., 2001; Tian et al., 2009; Zhang et al., 2002) with high spatiotemporal resolution. In *Drosophila* brain preparations, for example, the calcium sensor G-CaMP exhibits a fluorescence intensity change as large as 120% in specific antennal lobe glomeruli in response to odor stimulation (Wang et al., 2003). These techniques, however, do not easily permit the identification and marking of active neurons in a freely behaving animal. Immediate-early genes (IEGs) have historically been exploited to report neural activity for this purpose (Barth et al., 2004; Reijmers et al., 2007; Wang et al., 2006). One drawback associated with the use of current IEG-based reporters has been the difficulty in determining the cell type of labeled neurons.

Here we present a novel activity reporter system dubbed CaLexA (calcium-dependent nuclear import of LexA) that utilizes nuclear factor of activated T cells (NFAT), a transcription factor that is imported into the nucleus in an activity-dependent manner. Our rationale for selecting NFAT for the CaLexA system is based on three previous studies. First, NFAT in hippocampal neurons rapidly translocates from cytoplasm to the nucleus upon depolarization (Graef et al., 1999), suggesting that neurons have the necessary machinery to shuttle NFAT between the cytoplasm and nucleus. Second, the pathways regulating NFAT subcellular localization are conserved across different animal species, including mouse and *Drosophila* (Gwack et al., 2006). Third, sustained levels of intracellular calcium are required for NFAT-mediated transcription (Dolmetsch et al., 1997), suggesting that an NFAT-based reporter system may be unresponsive to low basal neural activity. We reasoned that these three features would make the NFAT-based neural tracing technique highly selective to specific neural activity and provide a system for circuit mapping.

**Figure 1:**
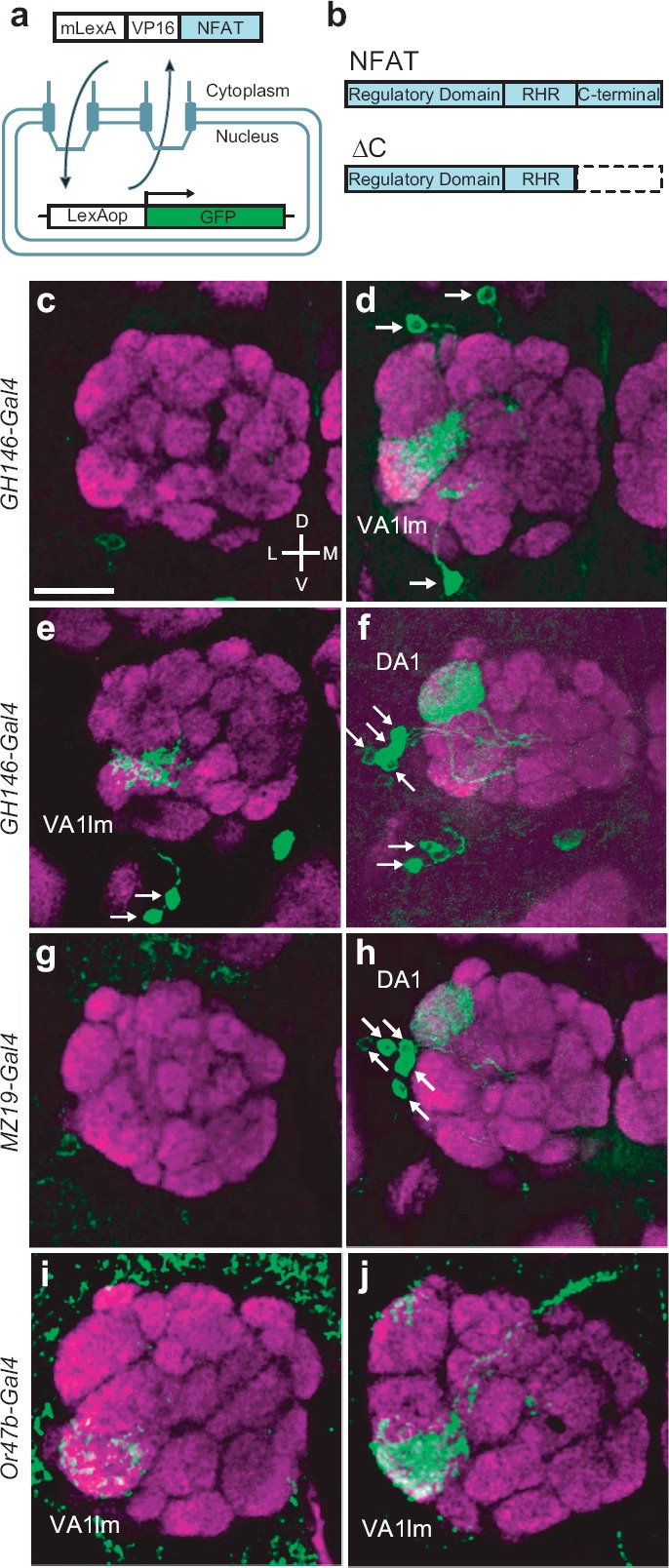
The CaLexA system and active antennal lobe neurons in response to fly odors, (**a**) Schematic illustration of the CaLexA system. Calcium accumulation activates calcineurin that dephosphorylates NFAT, causing the chimeric transcription factor mLexA-VP16-NFAT to shuttle into the nucleus. Once inside the nucleus, the chimeric transcription factor induces expression of the *GFP* reporter gene, which is under the control of the LexA operator (LexAop). (**b**) Schematic illustration of NFATc1 molecule that is used for the CaLexA system. NFAT comprises three major segments that include the regulatory, the RHR DNA binding, and the C-terminal domains. NFAT sequence without the C-terminal domain (ΔC) is used to make mLexA-VP16-NFAT. (**c-f**) Confocal images of the antennal lobe of flies bearing the *GH146-Gal4, UAS-mLexA-VP16-NFAT, LexAop-CD2-GFP,* and *LexAop-CD8-GFP-2A-CD8-GFP* transgenes. (**c, e**) Images of unstimulated flies, (**d**) Image of a male fly exposed to the odor of 10 virgin female flies, (**f**) Image of a female fly exposed to the odor of 10 male flies, (**g, h**) Confocal images of the antennal lobe of female flies bearing *MZ19-Gal4, UAS-mLexA-VP16-NFAT, LexAop-CD2-GFP,* and *LexAop-CD8-GFP-2A-CD8-GFP.* (**g**) Image of an unstimulated fly. (**h**) Image of a fly exposed to the odor of 10 male flies, (**i, j**) Confocal images of the antennal lobe of male flies bearing *Or47b-Gal4, UAS-mLexA-VP16-NFAT, LexAop-CD2-GFP,* and *LexAop-CD8-GFP-2A-CD8-GFP.* (**i**) Image of an unstimulated fly. (**j**) Image of a fly exposed to the odor of 10 virgin female flies. Whole-mount brain preparations were stained with anti-GFP (green) and nc82 (a marker of neuropil, magenta) antibodies. For all images, dorsal (top), ventral (bottom), lateral (left), medial (right). Arrows indicate cell bodies of PNs. Scale bar = 20 μm.

The CaLexA system targets a modified NFAT to specific neuronal populations. Its import into the nucleus upon sustained depolarization induces the expression of the reporter gene (Figure 1a and b). We employed two binary expression systems: the Gal4/UAS system (Brand & Perrimon, 1993) for expressing the chimeric transcription factor LexA-VP16-NFAT, and the LexA/LexAop system (Lai & Lee, 2006) for expressing the green fluorescent protein (GFP) reporter. The regulatory domain of NFATc1 contains the nuclear localization signal (NLS), whose function is tightly controlled by the calcium/calmodulin-dependent phosphatase calcineurin (Hogan et al., 2003). Both the mutant bacterial DNA-binding protein mLexA and the VP16 activation domain lack any NLS (La Boissiere et al., 1999; Rhee et al., 2000). Therefore, nuclear localization of the fusion protein mLexA-VP16-NFAT is controlled entirely by the NLS of NFAT; it should not enter the nucleus unless the cell experiences sustained depolarization. Repetitive exposure to a given sensory stimulation should therefore cause accumulation of the chimeric transcription factor in the nucleus of those neurons that process the sensory stimulation.

## METHODS

### DNA Construction

To generate mutant LexA-VP16 (mLexA-VP16), mutations in NLS were introduced by polymerase chain reaction (PCR), using primers LexA NLS mut up2 (CCCTTAACGGTAACCTCGTCATCAA) and LexA NLS mut down3 (CCTGGAAAAACAGGGCAATAAAGTCGAACT). To produce mLexA-VP16-NFAT construct, DNA fragments of mLexA-VP16 and human NFATc1 coding region were amplified by using primers LexA-VP16 up XhoI (ATAACTCGAGGGAATGAAAGCGTTAACGGCCA), LexA-VP16 down XbaI (ATAATCTAGACCCACCGTACTCGTCAATTC), NFATc1 up XbaI (AATATCTAGAATGCCAAGCACCAGCTTTCCAGT), and NFATc1 down SpeI (AATAACTAGTTTAGAAAAAGCACCCCACGCGCTC). Resultant mLexA-VP16 fragment was cloned into Xho I and Xba I sites of Bstm plasmid, then NFATc1 fragment was cloned into Xba I and Spe I sites. For DNA injection into fly embryo, PCR fragment with mLexA-VP16-NFAT was cloned into Not I and Spe I sites of pUAST plasmid. The following primers are used for PCR: LexA Not I up (AAAAGCGGCCGCGGAATGAAAGC GTTAACG) and NFATc1 down Spe I (AATAACTA GTTTAGAAAAAGCACCCCACGCGCTC). To produce NFAT deletion constructs, PCR fragments were amplified from plasmid with mLexA-VP16-NFAT and cloned into Not I and Spe I sites of pUAST by using following primers, LexA Not I up (AAAAGCGGCCGCGGAATGAAAGCGTTAACG), NFATc1 deletion down3 spe I (AATAACTAGTTTAAAGCTCATACGGGCCTGAGT), and NFATc1 C del down spe I (ATAACTAGTTCA GGAGCATTCGATGGGGTTGGA). To make deletion of calcineurin binding sites, PCR primers, NFATc1 cn1del up (ACTCTCCAGGGCAGGGGCCCCATCA) and NFATc1 cn1del down (TCGTGCTTGGGCCTGTACCACAACA A), were used. To produce LexAop-CD8-GFP-2A-CD8-GFP construct, DNA fragments with CD8-GFP-2A-CD8-GFP cloned into Bgl II and Xba I sites of pUAST were digested and cloned into pLot vector. Transgenic flies were generated by standard method.

### Construction of Vectors Used in In Vitro Experiments

A tetR-VP16 sequence is PCRed out from pUHG-15 (a gift from Dr. Hermann Bujard) and cloned into pcDNA3 by HindIII/BamHI. The hNFATc1(1–590) sequence is PCRed from pUAST-mLexA-VP16-hNFATc1 and cloned into pcDNA3-tetR-VP16 by BamHI/XhoI to produce tetR-VP16-GS-hNFATc1(1–590). The two sequences are linked with a GSG linker. A fragment of mNFATc2 sequence (accession no.: ACG55614.1) was cloned into pM by EcoRI/XbaI to produce Gal4-NFATAD(1–144). The firefly luciferase cDNA is PCRed from pFR-Luc (Stratagene) and cloned into pTRE-tight (Clontech) to produce tetO-Fluc.

### Cell-Based Transcription Assay

N2A cells were maintained in Dulbecco's modified Eagle's medium (DMEM) + 10% fetal bovine serum (FBS) following standard practice. Lipofectamine 2000 (Invitrogen, xx, xx) was used to transfect N2A cells with tetR-VP16-GS-hNFATc1, tetO-Fluc, and pRL-TK (Stratagene, xx, xx) at a ratio of 1:0.2:0.1. In case of potassium chloride stimulation, the KC1 concentration was raised to 60 mM at 24 hours post transfection. Gal4-NFATAD was transfected together with pcDNA3-VChR1-EYFP (a gift from Dr. Karl Deisseroth), pFR-Luc, and pRL-TK at a ratio of 1:0.2:0.2:0.1. We used a custom-made 488-nm LED (light emitting diode) array to deliver a 5-second-on/60-second-off patterned illumination at 24 hours post transfection for 12 hours. Luciferase assay were done at 48 hours post transfection with the Dual Luciferase Reporter system (Promega, xx, xx).

### Fly Strains

The following transgenic lines were used: (1) *GH146-Gal4* (Stocker et al., 1997), (2) *MZ19-Gal4* (Berdnik et al., 2006), (3) *LexAop-CD2-GFP* (Lai & Lee, 2006), (4) *fru*^GAL4^ (Stockinger et al., 2005), (5) *Or47b-Gal4* (Vosshall et al., 2000), (6) *Or83b-Gal4* (Wang et al., 2003), and (7) *LexAop-CD8-GFP-2A-CD8-GFP.*

### Immunohistochemistry

For immunohistochemistry, fly brains were dissected in calcium-free AHL saline (108 mM NaC1, 5mM KCL, 2 mM MgC1_2_, 4 mM NaHCO_3_, 1 mM NaH_2_PO_4_, 5 mM trehalose, 10 mM sucrose, 5 mM HEPES [pH 7.5]) and fixed with 4% paraformaldehyde in phosphate-buffered saline (PBS). After washed with PBST (PBS with 0.5% Triton X-100), brains were blocked using PBST with 1% bovine serum albumin (BSA). Rabbit anti-GFP (Invitrogen; 1:5000) and NC82 (University of Iowa Hybridoma Bank; 1:50) were used as primary antibodies, Alexa Fluor 488 anti-rabbit immunoglobulin G (IgG) (Invitrogen; 1:1000) and Alexa Fluor 568 anti-mouse IgG (Invitrogen; 1:1000) were used as secondary antibodies, respectively.

### Quantitative Analysis of GFP Fluorescence

For all fly odor experiments, male and virgin female flies were collected within several hours after eclosion and maintained separately. Odor exposure experiments were conducted in two chambers created by two small plastic tubes separated by a nylon mesh barrier and plugged with cotton caps at either end. A single fly in one chamber was exposed to the odor of either virgin female or week-old male CS flies placed in the second chamber. Fly brains were dissected in AHL saline. GFP fluorescence in the brains was analyzed with a custom-built two-photon microscope at the wavelength of 925 nm and quantified by ImageJ software.

### Ventral Nerve Cord (VNC) Analysis after Courtship Behavior

Male and wild-type virgin female flies (CS) were collected soon after eclosion and kept in separate food vials for 3–5 days. To examine neural activity patterns associated with courtship, one male fly and 10 virgin females were then put together in a tube. To control for neural activity patterns associated with female odors alone, one male and 10 virgin females flies were placed into a second tube where they were separated by a nylon mesh barrier. Male flies were dissected 24 hours later in AHL saline, and processed for immunohistochemistry.

## RESULTS

### Validation of the NFAT-Based Neural Tracing Method

To evaluate the accuracy and sensitivity of the CaLexA system, we first validated it against the well-characterized projection neurons (PNs) in the *Drosophila* olfactory system. Fly cuticular chemical extracts, upon contact with the antenna, preferentially excite olfactory receptor neurons that converge on the DA1 and VA1lm glomeruli (van der Goes van Naters & Carlson, 2007). Using the *GH146-Gal4* line, which labels 83 of about 150 PNs (Marin et al., 2002; Stocker et al., 1997; Wong et al., 2002), we expected to see GFP expression in DA1 and VA1lm in flies exposed to members of the opposite sex. Experiments were carried out in a small chamber with a nylon screen divider to prevent physical contact between male and female flies. NFAT sequence without the C-terminal domain (ΔC) is used to make UAS-mLexA-VP16-NFAT fly (Figure 1b). As predicted, male flies harboring *GH146-Gal4*, *UAS-mLexA-VP16-NFAT, LexAop-CD8-GFP-2A-CD8-GFP*, and *LexAop-CD2-GFP* showed robust fluorescence intensity in the VA1lm glomerulus after exposure to 10 female flies for 24 hours (Figure 1c and d). Female flies developed bright fluorescence intensity in the DA1 glomerulus (Figure 1e and f) after exposure to 10 male flies for 24 hours. These results are consistent with previous findings that neural activity in DA1 and VA1lm is important for courtship behavior in *Drosophila* (Dickson, 2008; Root et al., 2008). Unlike previous experiment that female odor was directly delivered to the antenna, male and female flies were separated to prevent direct contact in our experiments. Thus, this is the first demonstration that volatile female odor excite the VA1lm glomerulus.

We next asked whether the same glomeruli are labeled by the CaLexA system when used in combination with other Gal4 lines. The MZ19 line targets *Gal4* expression to PNs of the DA1, VA1d, and DC3 glomeruli (Berdnik et al., 2006). Consistent with the GH146 results, *MZ19-Gal4* female flies showed robust fluorescence intensity in the DA1 glomerulus after exposure to male flies (Figure 1g and h). Previous experiments have demonstrated that PN activity of a given glomerulus derives mostly from the cognate odorant receptor neurons (ORNs) (Olsen et al., 2007; Root et al., 2007). We thus applied the CaLexA system to investigate ORN activity in response to specific fly odor. Or47b ORNs converge onto the VA1lm glomerulus (Vosshall et al., 2000). After exposure to odor of virgin female flies, *Or47b-Gal4* male flies exhibit GFP expression in the VA1lm glomerulus (Figure 1i and j). The glomerulus-specific expression of the GFP reporter suggests that the mLexA-VP16-NFAT nuclear shuttling mechanism provides a novel approach for activity-dependent neural tracing.

**Figure 2:**
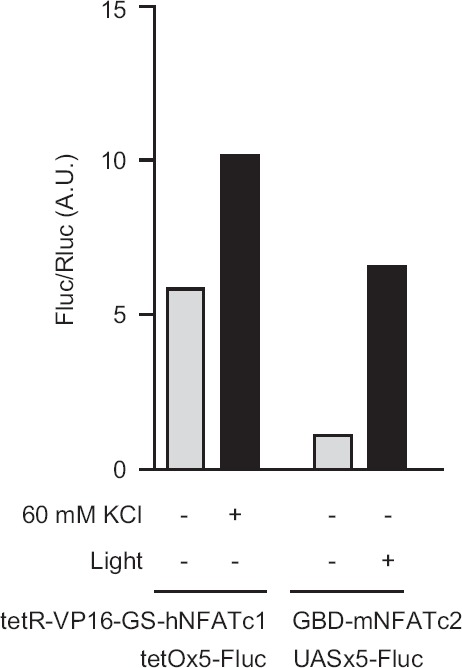
Activity-induced reporter gene expression in N2A cells. For KC1 stimulation experiments, tetR-VP16-GS-hNFAT was co-expressed with tetO-Fluc and pRL-TK. When stimulated with 60 mM KC1, the FLuc/RLuc ratio increased nearly 2-fold. For light stimulation experiments, Gal4-NFATAD was co-expressed with VChR1, UAS-Fluc (pFR-Luc), and pRL-TK. After 488-nm light illumination for 12 hours, the Fluc/Rluc ratio increased by about 5-fold. Each data point represents average results from two samples.

We have begun to investigate whether the CaLexA system for labeling active neurons can be adapted for mammalian system and whether neuronal activity induces reporter gene expression. We expressed two different NFAT fusion constructs in the neuroblastoma cell line N2A and measured expression levels of the reporter gene luciferase (Flue) induced by either high KC1 or light illumination in cells that simultaneously expressed channelrhodopsin. In response to 60 mM of KC1, N2A cells expressing *tetR-VP16-GS-hNFATc1* (1–590) and *tetO-Fluc* exhibited a nearly 2-fold increase in luciferase activity (Figure 2). In a different experiment, we fused the Gal4 DNA-binding fragment (GBD) with a mouse NFATc2 fragment. We showed that light illumination increased luciferase activity by 5-fold in N2A cells that expressed *VChR1, GBD-mNFATc2* (1–144), and *UAS-Fluc.* These experiments indicate that neuronal depolarization can indeed increase reporter gene expression in a mammalian cell line that expresses the NFAT-based constructs; however, further experiments are required to find the best expression system for mammals. Importantly, these proof-of-concept experiments suggest that CaLexA should work in the mammalian system as well.

### Quantification of Reporter Gene Expression in Response to Odor Stimulation

We next counted the number of GH146 PNs labeled under the CaLexA system in response to fly odors. The VA1lm PNs reside in two different locations in the antennal lobe. On average, we saw 2.0 ± 1.3 (mean ± *SD*; *n* = 8) PNs in the dorsal cluster and 1.6 ± 0.5 (*n* = 8) PNs in the ventral cluster. In response to male odor, female flies exhibited 4.3 ± 1.0 (*n* = 6) labeled PNs in the lateral cluster and 1.7 ± 0.5 (*n* = 6) PNs in the ventral cluster. The CaLexA system requires four different transgenic lines, the leaky expression of which could cause an overestimate of active PNs. It is therefore necessary to confirm that the labeled cells are GH146 PNs. Flies harboring *GH146-Gal4, UAS-mLexA-VP16-NFAT, UAS-CD8-RFP, LexAop-CD8-GFP-2A-CD8-GFP*, and *LexAop-CD2-GFP* should express RFP in all GH146 PNs and GFP in active GH146 PNs. After exposure to the male pheromone 11-*cis*-vaccenyl acetate (cVA), 6 PNs in the female flies expressed both GFP and red fluorescent protein (RFP), with 4 PNs in the lateral cluster and 2 PNs in the ventral cluster (Supplementary Figure 1; Supplementary Figures to be found online at http://www.informahealthcare.com/neg/doi/10.3109/01677063.2011.642910). We then tested the CaLexA system with the *MZ19-Gal4* line, which labels only DA1 PNs in the lateral cluster. Similarly, the exposure to cVA resulted in 5.5 ± 0.6 (*n* = 4) labeled PNs in the lateral cluster. These numbers of DA1 PNs are consistent with those in a previous report using a photoactivatable GFP (Datta et al., 2008). Furthermore, the consistent number of labeled DA1 PNs in the lateral cluster using two different Gal4 lines suggests that the CaLexA system provides a reliable method to visualize active DA1 PNs.

**Figure 3:**
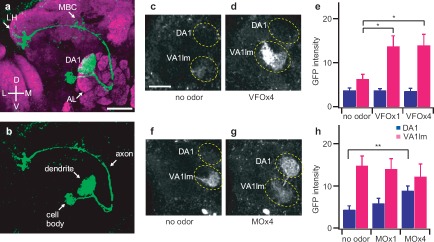
Quantitative analysis of reporter gene expression in response to fly pheromones. (**a**) Confocal image of the DA1 PNs in response to a male pheromone, cVA. The test fly contained the following transgenes: *MZ19-Gal4, UAS-mLexA-VP16-NFAT, LexAop-CD2-GFP,* and *LexAop-CD8-GFP-2A-CD8-GFP.* Whole-mount brain preparations were stained with anti-GFP (green) and nc82 antibodies (magenta). AL = antennal lobe; LH = lateral horn; MBC = mushroom body calyx, (**b**) Confocal image of the DA1 PNs in response to male pheromone cVA with only green channel, (**c, d**) Two-photon microscopic images of GFP fluorescence in the antennal lobe of male flies in response to the odor of virgin female flies. Male flies contain *GH146-Gal4, UAS-mLexA-VP16-NFAT, LexAop-CD2-GFP,* and *LexAop-CD8-GFP-2A-CD8-GFP.* (**e**) Measurement of GFP fluorescence intensity in DA1 and VA1lm of male flies in response to female odor, *n* = 9–12. VFOx1 and VFOx4 indicate odors from one and four wild-type virgin female flies, respectively, (**f, g**) Two-photon microscopic images of GFP fluorescence in the antennal lobe of female flies in response to male odor. Female flies contain *GH146-Gal4, UAS-mLexA-VP16-NFAT, LexAop-CD2-GFP,* and *LexAop-CD8-GFP-2A-CD8-GFP.* (**h**) Measurement of GFP fluorescence intensity in DA1 and VA1lm of female flies in response to male odor, *n* = 10–14. MO × 1 and MO × 4 indicate male odors from one and four wild-type flies, respectively. Odor exposure lasted for 24 hours. GFP fluorescence intensity is displayed in arbitrary unit. Error bars indicate standard error of mean. *p < 0.05; **p < 0.01. Wilcoxon signed-rank test. All measurements were obtained from a custom two-photon microscope with the same laser power (61 mW at the back aperture of the objective lens) at the wavelength of 925 nm. Scale bar = 20 μm.

For the purpose of circuit reconstruction, a neural tracing method that is capable of labeling all compartments of a neuron would be ideal and useful. The CaLexA system labeled DA1 PNs from dendrites to cell body and axon branches in a fly that was exposed to cVA (Figure 3a and 2b). The cell bodies of DA1 PNs reside in the lateral region of the antennal lobe and their axons course through the mushroom body and eventually innervate the ventral aspect of the lateral horn. To investigate the sensitivity of the CaLexA system, we next measured GFP fluorescence intensity in live brains with two-photon microscopy in the DA1 and VA1lm glomeruli in response to different concentrations of fly odors. The odor of one virgin female fly was sufficient to induce a detectable increase of fluorescence intensity in the VA1lm glomerulus of a male fly (Figure 3c–e). The odor of four mature male flies was sufficient to generate detectable fluorescence intensity in the DA1 glomerulus of a female fly (Figure 3f–h). It is interesting to note that female flies exhibited higher background fluorescence intensity in VA1lm than male flies. This may reflect a response to self odor and the high concentration of female sex pheromone in virgin female flies soon after eclosion. In contrast, this background fluorescence may be absent in males because male sex pheromone levels do not peak until day 7 after eclosion (Bartelt et al., 1985).

We next compared the CaLexA system with calcium imaging. Previous experiments with calcium imaging show that increasing the concentration of cider vinegar activates more glomeruli (Semmelhack & Wang, 2009). The *Or83b-Gal4* line labels most of the ORNs, with which we can compare glomerular patterns in response to different concentrations of cider vinegar. Under the CaLexA system, exposure to a higher concentration of cider vinegar resulted in more intense expression of GFP in the same glomeruli and more glomeruli expressing GFP (Supplementary Figure 2 to be found online at http://www.mfor-mahealthcare.com/neg/doi/10.3109/01677063.2011.642910). How does the sensitivity of the CaLexA system compare to other neural recording techniques? Previous electrical recording studies in *Drosophila* demonstrated that the Or47b ORNs respond to high concentrations of female cuticular extracts when directly applied to the sensory neurons (van der Goes van Naters & Carlson, 2007). Our experiments with the CaLexA system provide the first evidence that a single fly emits sufficient amounts of volatile pheromones to activate Or47b neurons.

### Optimization of the CaLexA Neural Tracing Method

**Figure 4:**
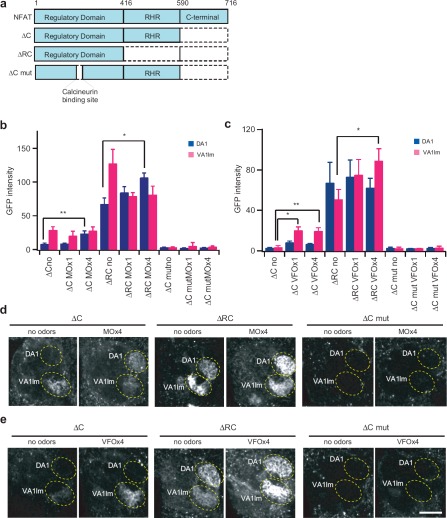
Comparison of different NFATc1 fragments for the CaLexA system, (**a**) The NFATc1 protein is comprised of three major segments that include the regulatory (aa 1–416), RHR DNA binding (aa 416–590), and C-terminal domains (aa 590–716). Different segments of the NFAT molecule were used to make the chimeric transcription factor for the CaLexA system. ΔC lacks the C-terminal; ARC lacks the RHR domain and the C-terminal. The regulatory domain contains calcineurin binding site. Six amino acids, PRIEIT, were deleted in the ΔC mutant construct, (**b**) GFP fluorescence intensity in DA1 and VA1lm of female flies in response to male odor. Female flies contain the *GH146-Gal4, LexAop-CD2-GFP,* and *UAS-mLexA-VP16-NFAT* transgenes. The ΔC version has the least background and best signal-to-noise ratio. MO × 1 and MO × 4 indicate male odor from one and four wild-type flies, respectively. *n* = 3–12. (**c**) GFP fluorescence intensity in DA1 and VA1lm of male flies in response to female odor. Male flies contain the *GH146-Gal4, LexAop-CD2-GFP,* and *UAS-mLexA-VP16-NFAT* transgenes. VFO × 1 and VFO × 4 indicate odor from one and four wild-type virgin female flies, respectively, *n* = 3–10. (**d, e**) Two-photon microscopic images of GFP fluorescence in the antennal lobe, (**d**) Images of neural activity patterns in the female fly brain after exposure to the male odor, (**e**) Images of neural activity patterns in the male fly brain after exposure to the female odor. Error bars indicate standard error of mean. *p < 0.05; **p < 0.01. Wilcoxon signed-rank test. All measurements were obtained from a custom two-photon microscope with the same laser power (86 mW at the back aperture of the objective lens) at the wavelength of 925 nm.

We investigated which NFAT fragment is appropriate for the activity reporter system. The NFAT molecule comprises three major domains (Hogan et al., 2003). The regulatory domain contains calcineurin binding sites, numerous phosphorylation sites, NLS, and nuclear export signal. Dephosphorylation by calcineurin unmasks the NLS, triggering nuclear translocation of NFAT. Although the RHR (Rel Homology Region) DNA binding and the C-terminal domains are apparently not required for the CaLexA system in activity-dependent neural tracing, it is possible that the mere size of the fusion protein would affect the efficacy of gene transcription. Expression of full-length NFAT seemed to cause lethality in *Drosophila* (data not shown). Therefore, we systematically deleted different NFAT domains to search for a better molecular design for the CaLexA system (Figure 4). Our results from sex pheromone-exposed flies expressing *GH146-Gal4, UAS-mLexA-VP16-NFAT,* and *LexAop-CD2-GFP* show that deleting both the RHR and C domains raises the baseline fluorescence intensity in the corresponding glomeruli (Figure 4b–e). For this sensory stimulation condition, the NFAT fragment with C domain deletion yields the largest fractional changes from control. Furthermore, flies expressing the ΔC NFAT fragment in ORNs or PNs do not appear to have any associated lethality (data not shown). We also investigated whether reporter gene expression is under the control of neural activity via calcineurin. In flies expressing a mutant NFAT that lacks the calcineurin binding sites, the same sensory stimulation failed to generate detectable fluorescence intensity in any glomeruli. These experiments suggest that the regulatory domain is sufficient to provide the molecular machinery for activity-dependent neural tracing in the CaLexA system.

**Figure 5:**
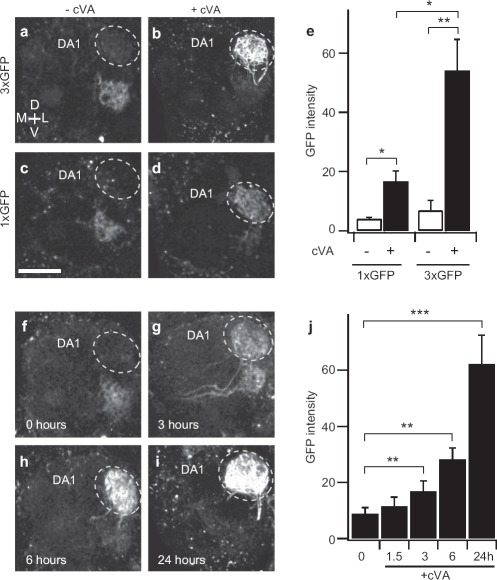
Increasing reporter gene copy number improves sensitivity of the CaLexA system, (**a–e**) Measurement of GFP fluorescence intensity in DA1 of female flies after exposed to cVA (20 μg) for 24 hours. The test fly contained *GH146-Gal4, UAS-mLexA-VP16-NFAT,* and *LexAop-CD2-GFP* (1 × GFP) or both *LexAop-CD2-GFP* and *LexAop-CD8-GFP-2A-CD8-GFP* (3 × GFP). (**a–d**) Images of GFP fluorescence in antennal lobe, (**e**) Quantitative analysis of GFP fluorescence in DA1. (**f–i**) Images of GFP fluorescence in antennal lobe, *n* = 6–9. (**j**) Quantitative analysis of GFP fluorescence in DA1. GFP fluorescence intensity is displayed in arbitrary unit, *n* = 6–9. Error bars indicate standard error of mean. *p < 0.05; **p < 0.01; ***p < 0.001. Wilcoxon signed-rank test. All measurements were obtained from a custom two-photon microscope with the same laser power (61 mW at the back aperture of the objective lens) at the wavelength of 925 nm. Scale bar = 20 μm.

It is important to obtain high signal-to-noise ratio for reliable neural tracing. Nuclear localization of NFAT is transient, because there are multiple NFAT kinases to phosphorylate NFAT for nuclear export (Gwack et al., 2006). In this context, increasing the copy number of the GFP gene should result in more robust reporter gene expression. Indeed, female flies bearing the *LexAop-CD8-GFP-2A-CD8-GFP* and *LexAop-CD2-GFP* exhibited about 8 times more GFP fluorescence in DA1 when exposed to cVA for 24 hours than that of control flies without cVA exposure (Figure 5a, b, and e). In contrast, female flies with just one copy of the *LexAop-CD2-GFP* transgene showed only 4 times the GFP level (Figure 5c, d, and e). With three copies of the reporter gene, exposure to cVA for as short as 3 hours yielded detectable GFP increase in DA1 (Figure 5f–j). Therefore, increasing GFP reporter gene copy number is a practical way to improve the sensitivity of the CaLexA neural tracing method.

**Figure 6:**
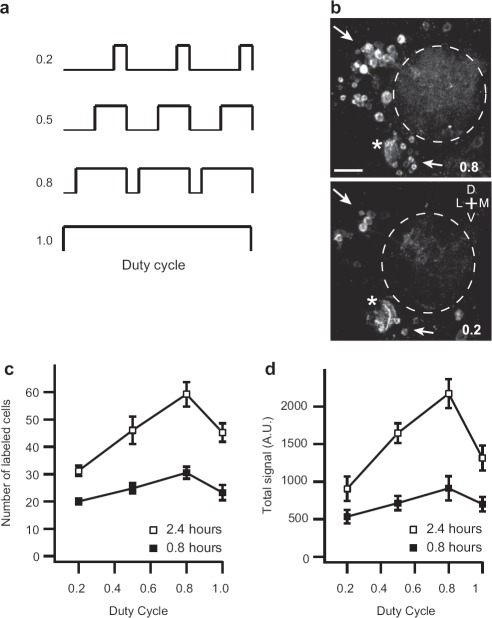
Analysis of reporter gene expression with different stimulus protocols in response to CO_2_. (**a**) Stimulus protocols of varying duty cycles. Pulsed CO_2_ (25 mL/min, 160 seconds/cycle) was mixed into continuous airflow (50 mL/min). The total duration of CO_2_ exposure was either 0.8 or 2.4 hours, (**b**) Two-photon microscopy images of GFP fluorescence in antennal lobe. Analysis was performed at 12 hours after the start of CO_2_ exposure. Arrows indicate labeled cell bodies in flies with exposure to CO_2_ at different duty cycle, 0.8 (top) or 0.2 (bottom). Asterisks: antennal nerve. Dotted lines: antennal lobe. The test fly contained *Elav-Gal4, UAS-mLexA-VP16-NFAT, LexAop-CD2-GFP,* and *LexAop-CD8-GFP-2A-CD8-GFP.* (**c, d**) Total number (**c**) and total fluorescence (**d**) of labeled cells in flies exposed to CO_2_ for 0.8 (filled box) and 2.4 (opened box) hours at different duty cycles, *n* = 4. Error bars indicate standard error of the mean. All measurements were obtained from a custom two-photon microscope with the same laser power (86 mW at the back aperture of the objective lens) at the wavelength of 925 nm. Scale bar = 20 μm.

We next investigated the relationship between stimulus patterns and reporter gene expression. Pulsed CO_2_ was mixed with continuous airflow to generate different stimulation patterns ranging from 20% to 100% duty cycle (Figure 6a). Total exposure duration was fixed for each condition. Many neurons in the antennal lobe, most of which are local interneurons, exhibited GFP expression when flies were exposed to CO_2_ (Figure 6b). The number of labeled neurons and the total fluorescence intensity increased with the duty cycle up to 80% but decreased thereafter when CO_2_ was continuously applied (Figure 6c and d). These results indicate that reporter gene expression in the CaLexA system is most sensitive to sustained but not continuous neural activity.

### Comparison of GFP Signal Intensity in Cell Bodies and Dendrites

**Figure 7:**
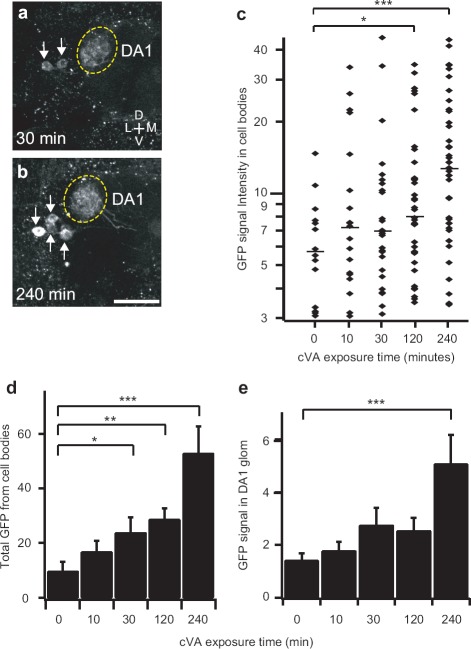
Quantitative analysis of reporter gene expression in PN cell bodies and dendrites. (**a, b**) Two-photon microscopy images of GFP fluorescence in antennal lobe. Arrows indicate PN cell bodies. Flies were exposed to cVA (20 μg) for 30 (**a**) or 240 (**b**) minutes. Images were obtained at 240 minutes. Results were from male flies containing *MZ19-Gal4, UAS-mLexA-VP16-NFAT, LexAop-CD2-GFP,* and *LexAop-CD8-GFP-2A-CD8-GFP.* (**c-e**) Analysis of GFP fluorescence intensity in cell bodies and dendrites of the DA1 PNs in flies exposed to cVA for different durations. Measurements for each exposure duration were obtained from 12 antennal lobes, (**c**) GFP fluorescence in the cell bodies. Horizontal bars: median values, (**d**) Total GFP fluorescence from all labeled cell bodies in each antennal lobe, (**e**) GFP fluorescence intensity (arbitrary unit) in PN dendrites of the DA1 glomerulus. Error bars indicate standard error of mean. *p < 0.05; **p < 0.01; ***p < 0.001. Wilcoxon signed-rank test. All measurements were obtained from a custom two-photon microscope with the same laser power (86 mW at the back aperture of the objective lens) at the wavelength of 925 nm. Scale bar = 20 μm.

Since the reporter protein GFP is synthesized in the cell bodies, it may take additional time for GFP molecules to travel to other cellular compartments. We therefore analyzed GFP fluorescence in PN cell bodies and dendrites in response to cVA. The *GH146-Gal4* line contains two populations of PNs that innervate the DA1 glomerulus, whereas the *MZ19-Gal4* line labels only the lateral DA1 PNs. Using the *MZ19-Gal4* line should therefore allow us to compare the fluorescence signal of the cell bodies and dendrites in a uniform population of neurons. In this experiment, we measured fluorescence intensity in the DA1 PNs of young male flies exposed to cVA for varying lengths of time. Longer time exposures resulted in more labeled cells and higher fluorescence intensities in each labeled cell body (Figure 7a and b). In male flies exposed to cVA for 30 minutes, 26% of the lateral DA1 PNs exhibited detectable fluorescence intensities ranging from 3.1 to 45.0 units with a median of 6.9. In males exposed to cVA for 240 minutes, however, 58% of the lateral DA1 PNs showed fluorescence intensities ranging from 3.5 to 44.1 units, with a median of 12.7 (Figure 7c). Total GFP intensity of all labeled cell bodies was significantly detected with 30 minutes exposure to cVA (Figure 7d). However, fluorescence intensity at dendrites in the DA1 glomerulus was significantly detected only after exposure to cVA for at least 240 minutes (Figure 7e). These results suggest that membrane-tethered GFP molecules take time to diffuse into dendrites. Fluorescence measurement in cell bodies appears to be a faster report of neural activity.

### Visualizing Neural Circuits Underlying Complex Behavior That Require Multimodal Sensory Inputs

The principal value of an activity-dependent reporting system lies in its ability to quickly identify key neural circuits for complex behaviors. Courting male flies exhibit a complex sequence of stereotyped behaviors (e.g., orienting towards the female, following/chasing her, wing vibrations, licking her genitalia, attempted copulation, and copulation) that are dependent upon the male's ability to detect female pheromone (Greenspan & Ferveur, 2000; Jallon, 1984). In addition to a rich behavioral output, multiple sensory inputs (e.g., visual, gustatory, auditory, mechanosensory) are received during the courting and mating process (Markow, 1987).

**Figure 8:**
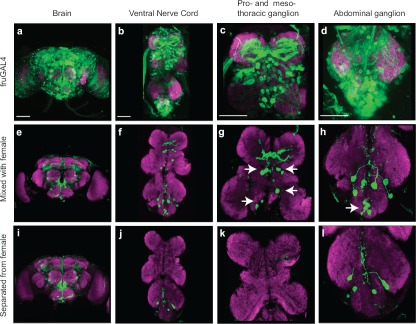
Visualizing neural circuits underlying complex behavior, (**a–d**) Expression pattern of *fru*^GAL4^ in the brain and ventral nerve cord (VNC). The male fly contained *fru*^GAL4^ and *UAS-CD8-GFP.* (**a**) Expression of *fru*^GAL4^ in the brain, (**b**) Expression of *fru*^GAL4^ in the VNC. (**c**) Expression of *fru*^GAL4^ in the prothoracic ganglion and mesothoracic ganglion, (**d**) Expression of *fru*^GAL4^ in the abdominal ganglion, (**e–h**) GFP expression in the brain and VNC of a male fly that was mixed with virgin female flies. The test male fly contained *fru*^GAL4^, *UAS-mLexA-VP16-NFAT, LexAop-CD2-GFP* and *LexAop-CD8-GFP-2A-CD8-GFP.* (**i–l**) GFP expression in the brain and VNC of a male fly that was separated from virgin female flies by a nylon mesh. Whole-mount brain and VNC preparations were stained with anti-GFP (green) and nc82 (a marker of neuropil, magenta) antibodies. Arrows indicate the labeled cells that were correlated with courtship behavior. Scale bar = 50 μm.

We used the CaLexA system to visualize neural circuits that mediate complex courtship behavior in *Drosophila* by targeting the *mLexA-VP16-NFAT* transgene to neurons involved in a sexually dimorphic circuit. The male-specific *fruitless (fru)* gene product (Fru^M^) plays a critical role in male courtship behavior (Baker et al., 2001). We thus limited expression of the *mLexA-VP16-NFAT* transgene to Fru^M^-expressing neurons using *fru*^GAL4^ (Figure 8a–d) (Stockinger et al., 2005). A male fly housed in a small chamber with 10 virgin female flies normally displays all the stereotyped courtship behaviors. However, separation of the male fly from female flies with a nylon screen abolished most of the courtship behaviors despite the availability of some sensory inputs such as olfactory information (Ejima et al., 2005). The patterns of labeled cells in both conditions were much sparser than that of Fru^M^-expressing neurons. This may be caused by low expression levels of the *mLexA-VP16-NFAT* transgene in some neurons. Therefore, pattern difference between the two behavior conditions may not be fully visible with the CaLexA system. The patterns of GFP-labeled cells in the brain appeared to be similar between the two conditions. Interestingly, GFP fluorescence label was detected in numerous cells in the mesothoracic ganglion and abdominal ganglion of the ventral nerve cord (VNC) in the male flies with all sensory information (Figure 8e–h), but absent in the male flies with limited sensory information (Figure 8i–l). These results show that the CaLexA system is capable of visualizing key neural circuits for complex behaviors.

## DISCUSSION

Robust but simple neural techniques to visualize active neurons in behaving animals are an important tool to investigate the link between neural circuits and behaviors. Immunostaining for IEGs and IEG-based reporter gene expression systems have been used in mammals to label active neurons (Barth et al., 2004; Reijmers et al., 2007; Wang et al., 2006). In *Drosophila,* levels of synaptic calmodulin (CaM) kinase II expression have been exploited to report neural activity (Ashraf et al., 2006). The CaLexA system is, to our knowledge, the first time NFAT has ever been used in this context and the first report of a robust activity-dependent neural circuit tracer in *Drosophila.*

The key element in our CaLexA system is the chimeric transcription factor made from the calcineurin-dependent NLS of NFAT and a mutant LexA transcription factor that lacks NLS. Reporter gene expression provides a metric of neural activity, because it controls nuclear import of the fusion transcription factor. In the proof-of-concept experiments, we restricted expression of the transcription factor to ORNs or PNs in *Drosophila* and examined which neurons selectively respond to the fly pheromones. We showed that male fly pheromone excites the DA1 glomerulus and female pheromone excites the VA1lm glomerulus. This reporter system is able to label all cellular compartments of active cells from dendrites to cell body and axon terminals. Furthermore, we have begun to use the CaLexA system to visualize neural circuits underlying complex behaviors. Our CaLexA system appears to have improved selectivity and signal-to-noise ratio over several similar systems using the IEGs.

This approach validated in *Drosophila* should, in principle, be applicable to other model organisms with some modifications. For the CaLexA system in its current form, we made use of the Gal4/UAS system to express the genetically engineered transcription factor mLexA-VP16-NFAT. Whereas the binary expression system Gal4/UAS has been used in the zebra fish (Davison et al., 2007; Scott et al., 2007) and mice (Ornitz et al., 1991), the tTA/tetO and Cre/LoxP expression systems are more popular in mice (Luo et al., 2008). For the CaLexA system to work in mice and take advantage of the existing mouse lines with reporter genes in tetO or LoxP forms, one will need to create a mouse with the appropriate promoter to control the expression of NFAT-tTA in specific cell populations, or to generate a viral vector with NFAT-tTA. Whether the modified NFAT will compete with endogenous NFAT in mammalian systems for calcineurin needs to be determined empirically. However G-CaMP, a genetically expressed calcium indicator, does not seem to compete with endogenous calcium-binding proteins for calcium to cause abnormal physiology or behavior (Jayaraman & Laurent, 2007; Tian et al., 2009). Our preliminary results in a mammalian cell line suggest that the CaLexA tracing technique is poised to work in mammals.

The CaLexA system is well suited for visualizing neural activity in intact animals on long but not short time scales in its current form. This technique could be useful in visualizing neuromodulation by internal physiological states (Dacks et al., 2009; Root et al., 2011). Although imaging neural activity in behaving animals is possible, technical hurdles make it difficult to study multisensory integration. We have applied the CaLexA system to visualize active neurons in complex behaviors. Separation of male flies from the mating partners by a porous nylon screen abolished most courtship behaviors. In this experiment, we found a small subset of Fru^M^-expressing neurons in the VNC was correlated with courtship activity. This result supports the notion that neural activity in Fru^M^-expressing neuron contributes to male courtship behaviors. One important step in male courtship behavior is song production, which requires multimodal inputs and male-specific neurons in the VNC (Antony & Jallon, 1982; Clyne & Miesenbock, 2008; Rideout et al., 2010). Our study showed that non-contact olfactory cue elicited very little neural activity in Fru^M^-expressing neurons in the VNC. Furthermore, these results are consistent with the notion that integration of multiple sensory inputs activates specific neurons in the VNC to enable courtship behaviors. Alternatively, the labeled cells in the VNC may also include motor neurons that are active during the mating or courting process. Future experiments to investigate the function and behavioral relevance of these labeled neurons will provide an entry point to unravel the neural circuit underlying courtship song production.
