# Supplementary material for: Mapping Neural Circuits with Activity-Dependent Nuclear Import of a Transcription Factor
Source: J Neurogenet. 2012 Jan 12;26(1):89–102. doi: 10.3109/01677063.2011.642910 (PMC3357894; doi:10.3109/01677063.2011.642910)
Supplement: Supplementary file 1 [file gneg26-89-SD1.pdf]

Supplementary materials for Masuyama K, Zhang Y, Rao Y, Wang J W. Mapping Neural Circuits with Activity-Dependent Nuclear Import of a Transcription Factor, *J. Neurogenetics*, 2012; 26(1): 89–102.

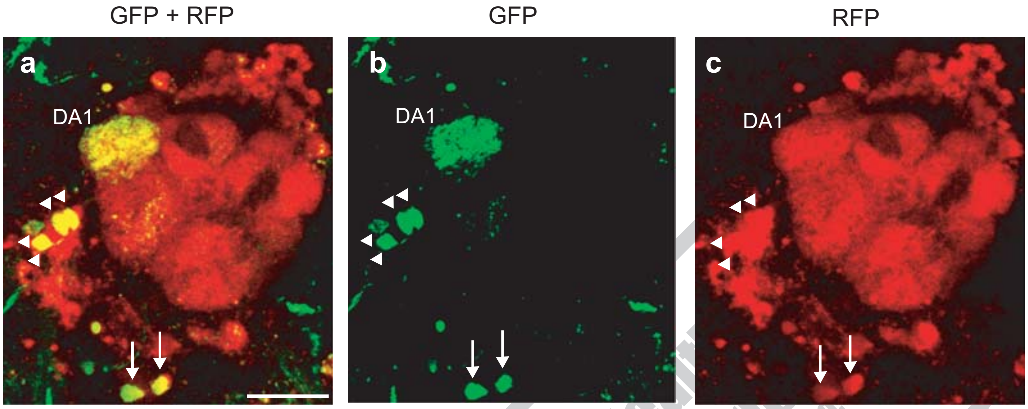

Supplementary Figure 1. Visualizing active PNs with GFP and all GH146 PNs with RFP. (**a**) Confocal image of the antennal lobe of a fly bearing the *GH146-Gal4*, *UAS-mLexA-VP16-NFAT*, *UAS-CD8-RFP*, *LexAop-CD2-GFP*, and *LexAop-CD8-GFP-2A-CD8-GFP* transgenes. After exposed to cVA for 24 hours, GFP is expressed in the DA1 PNs (green color) of a test fly. All GH146 PNs express RFP (red color). (**b**) Confocal image same as **a**, with only the green channel. (**c**) Confocal image same as **a**, with only the red channel. Arrows indicate PN cell bodies in the ventral cluster. Arrow heads indicate PN cell bodies in the lateral cluster. Scale bar = 20 µm.

Copyright © 2012 Informa Healthcare
Not for Sale or Commercial Distribution
Unauthorised use prohibited. Authorised users can download,
display, view and print a single copy for personal use

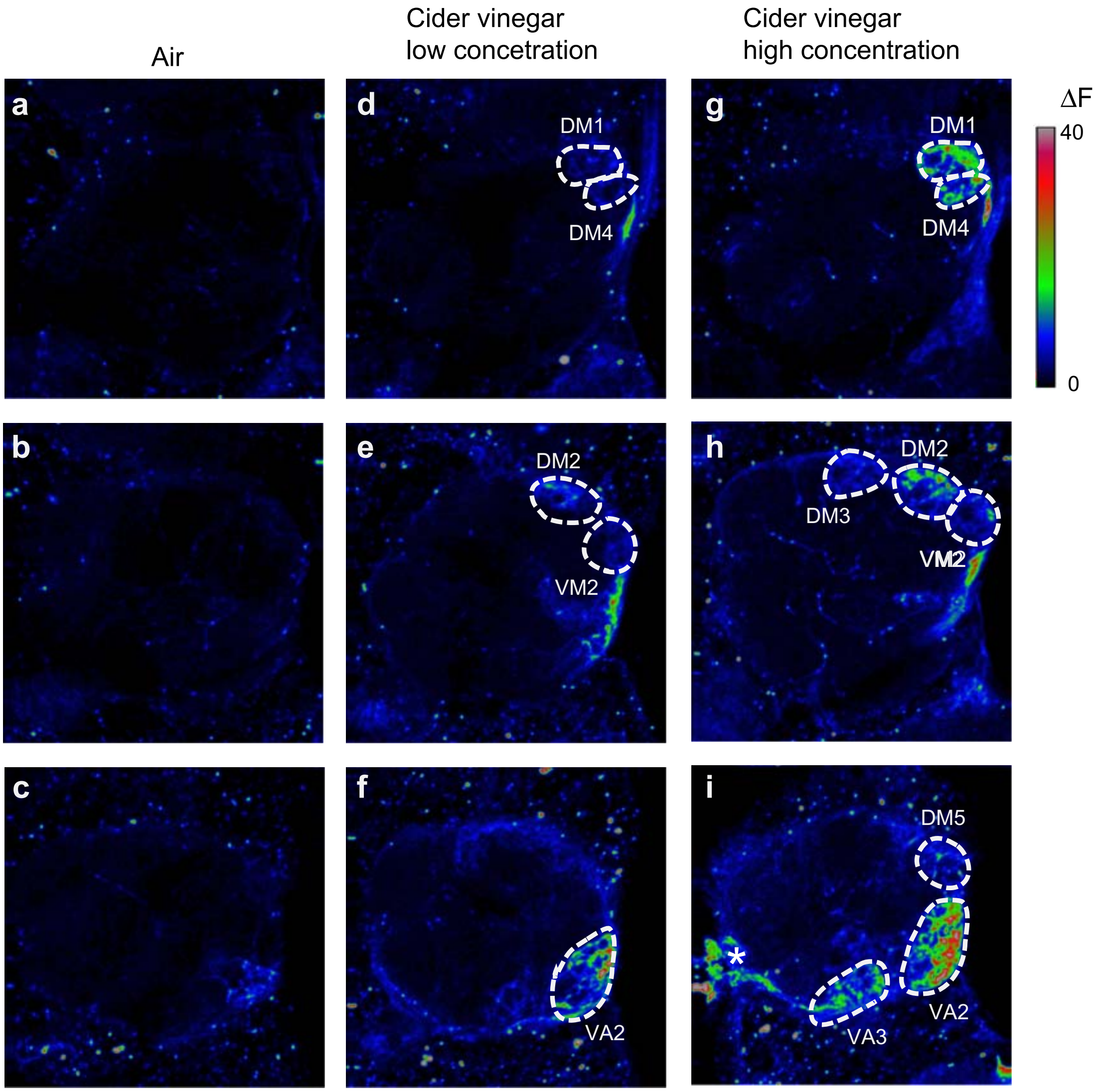

Supplementary Figure 2. Glomerular pattern in response to cider vinegar at different concentrations. A higher concentration of cider vinegar excites more glomeruli and induces more GFP expression in the same glomerulus. GFP fluorescence in antennal lobe detected by two-photon microscopy is shown as pseudocolored images. Male flies bearing the *Or83b-Gal4*, *UAS-mLexA-VP16-NFAT*, *LexAop-CD2-GFP*, and *LexAop-CD8-GFP-2A-CD8-GFP* transgenes were exposed to air (500 mL/min; **a**–**c**), low concentration (83 mL/min; **d**–**f**), or high concentration of cider vinegar (500 mL/min; **g**–**i**) for 24 hours.
